# Rescue extracorporeal life support as a bridge to durable left ventricular assist device

**DOI:** 10.1177/03913988211053874

**Published:** 2021-10-21

**Authors:** Alina Zubarevich, Konstantin Zhigalov, Marcin Szczechowicz, Arian Arjomandi Rad, Robert Vardanyan, Saeed Torabi, Maria Papathanasiou, Peter Luedike, Achim Koch, Nikolaus Pizanis, Markus Kamler, Bastian Schmack, Arjang Ruhparwar, Alexander Weymann

**Affiliations:** 1 Department of Thoracic and Cardiovascular Surgery, West German Heart and Vascular Center, University of Duisburg-Essen, Essen, Germany; 2 Department of Medicine, Faculty of Medicine, Imperial College London, London, UK; 3 Department of Anesthesiology and Intensive Care Medicine, University Hospital of Cologne, Cologne, Germany; 4 Department of Cardiology & Vascular Medicine, West German Heart and Vascular Center, University of Duisburg-Essen, Essen, Germany

**Keywords:** Rescue ECLS, durable LVAD, cardiogenic shock, ECPELLA, Levitronix

## Abstract

**Background:**

The ideal timing of a durable assist device implantation in patients with end-stage heart failure presenting with INTERMACS profile I is still controversial. The data on extracorporeal life support (ECLS) bridge to durable left ventricular assist device (LVAD) in these patients is limited.

**Materials and methods:**

We retrospectively analyzed the outcomes of 35 patients in acute cardiogenic shock (CS) who, between December 2013 and September 2020, were bridged with ECLS to durable LVAD. The mean age was 52.3 ± 12.0 years. The primary endpoints of this study were in-hospital, 30-day, 6-month, and 1-year mortality. The secondary endpoint was the development of any postoperative adverse events and other characteristics during the follow-up period. We also assessed the impact of the rescue ECLS on the recovery of the end-organ function.

**Results:**

In-hospital, 30-day, 6-month, and 1-year survival was 65.6%, 75.9%, 69.2%, and 62.7% respectively. The median time on ECLS was 7 days (IQR 5.0–13.0). We observed a high incidence of a severe right heart failure (22.9%), acute kidney injury on dialysis (68.6%), and respiratory failure (77.1%). Bridge with ECLS provided a significant recovery of liver and kidney function prior to durable LVAD implantation.

**Conclusion:**

The concept of bridging patients presenting in end-stage heart failure and cardiogenic shock with ECLS prior to durable LVAD implantation is a feasible method to ensure acceptable survival rates and significant recovery of the end-organ function.

## Introduction

With the incremental development of expertise and knowledge in the field of surgical heart failure (HF) therapy, the overall mortality remains inadmissibly high, particularly in patients admitted in INTERMACS profile I and II, as according to the current classification of Interagency Registry for Mechanically Assisted Circulatory Support (INTERMACS).^1,2^ Whilst the ideal implant time of a durable assist device in patients with end-stage heart failure is considered prior to their development of cardiogenic shock (CS), the gold standard treatment of patients that present with refractory CS remains controversial.^3^ In the annual INTERMACS report, the authors describe that prior to durable left-ventricular assist device (LVAD) implantation, 50% of the patients presented with INTERMACS profile I or II.^4^

Moreover, whilst Molina et al.^4^ report a significant improvement in survival and a decrease in periprocedural complications, LVAD implantation in patients presenting with CS is still associated with a high risk of right heart failure (RHF), cerebrovascular events, infection, pump thrombosis, and hemolysis. However, in times of extreme donor organ shortage, an expansion of the indications for durable LVAD implantation into the high-risk group of patients presenting with CS may be the only therapeutic option for this specific cohort. As a result, we are observing the increasing number of ultima ratio LVAD implantations in high-risk patients.

In this study, we review our experience with the rescue extracorporeal life support (ECLS) as a bridge to durable LVAD implantation in patients presenting with acute CS and INTERMACS profile I. This cohort of patients presents with particularly high morbidity and mortality.^5,6^ ECLS helps to stabilize the patients’ hemodynamics, serves the stabilization of the end-organ function, and helps in transferring the patients to a more favorable INTERMACS stage prior to durable LVAD implantation.^7^

The aim of this study is to investigate the impact of rescue ECLS implantation on the further durable implantation in patients presenting with CS unrelated to postcardiotomy syndrome.

## Materials and methods

### Study population

At our institution, between December 2013 and September 2020, a total of 35 patients presenting with CS and INTERMACS profile I underwent rescue ECLS implantation as a bridge to durable LVAD. The indications for the procedure were made according to the current guidelines for the diagnosis and treatment of acute and chronic heart failure.^8^ We used two LVAD devices: HeartMate III (HM III, Thoratec Corp., Pleasanton, CA, USA) and HeartWare (HVAD, HeartWare International Inc. Framingham, MA, USA). The choice of LVAD model was based on the availability of the devices in the clinic and the surgeons’ personal preference.

### Study design

The study is a retrospective review of prospectively collected data. Data collected as part of the institutional Mechanical Circulatory Support Database included detailed information on patient demographics; baseline clinical characteristics; laboratory, echocardiographic and hemodynamic parameters; and other intraoperative variables and postoperative outcomes. The follow-up data was collected at planned periodic presentations of patients at our VAD clinic. The study was approved by the local ethics committee (20-9283-BO).

### Outcome measures

The primary endpoints of this study were in-hospital, 30-day, 6-month, and 1-year mortality. The secondary endpoint was the development of any postoperative adverse events and other characteristics during the follow-up period. Additionally, we assessed the impact of the rescue ECLS on the recovery of the end-organ function.

### Variables and definitions

Variables were evaluated, including baseline characteristics, as well as further preoperative clinical data, preoperative laboratory parameters, intraoperative data, postoperative variables, and follow-up data. The adverse events definitions were mostly based on the “INTERMACS Adverse Event Definitions.”^2^ *Major bleeding* was defined as an episode of suspected internal or external bleeding that resulted in re-operation, hospitalization, or transfusion of packed red blood cells (PRBCs) as follows: ⩾4 U PRBC within any 24-h period during first 7 days post-implant or a transfusion of PRBC after 7 days following implant. *Major infection* was defined as an episode of localized non-device infection, percutaneous site and/or pocket infection, or sepsis. *Respiratory failure* was defined as an impairment of respiratory function requiring reintubation and/or tracheostomy, or the inability to discontinue ventilator support within 6 days (144 h) post-LVAD. *RHF* was defined as a need for post-implant inotropes continued beyond post-op day 14 following LVAD implantation, right ventricular assist device at any time following LVAD implantation, or delayed chest closure due to hemodynamic instability. *Hepatic dysfunction* was defined as an episode of an increase in any two of the following hepatic laboratory values; total bilirubin, aspartate aminotransferase (AST), and alanine aminotransferase (ALT) to a level greater than three times the upper limit of normal, beyond 14 days post-implantation. *Acute renal dysfunction* was defined as an episode of abnormal kidney function requiring dialysis in patients who did not require this procedure prior to implantation, or a rise in serum creatinine of greater than three times the baseline or greater than 5 mg/dl sustained for over 48 h. *Neurological dysfunction* was defined as an episode of transient ischemic attack, ischemic stroke, or acute intracranial hemorrhage.

### Surgical technique

The cannulation for central ECSL was performed via the ascending aorta and right atrium. The aortic cannulation was performed either directly in the ascending aorta or in “chimney” technique, where an 8 mm vascular graft was anastomosed to the ascending aorta and percutaneously tunneled under the xyphoid. The peripheral ECLS implantation was performed via direct percutaneous cannulation of the femoral artery or right subclavian artery via a vascular graft and percutaneous cannulation of a femoral vein. In some cases, ECLS has been switched to the short-term LVAD (Levitronix CentriMag; Thoratec) via median sternotomy with percutaneous cannulas prior to durable LVAD implantation, as described previously by Zeriouh et al.^9^ All LVAD procedures were performed via median sternotomy. After systemic heparinization, cannulation for CPB was performed either via ascending aorta and right atrium or the procedure was performed on ECLS, without conversion to CPB. The concomitant procedures were performed prior to the LVAD implantation in cardioplegic arrest. The inflow LVAD was implanted into the apex cordis according to the manufacturer’s instructions. The outflow graft was connected to the ascending aorta via an end-to-side anastomosis with 5.0 prolene running sutures. The driveline was undermined subcutaneously in a double-tunnel technique. After weaning the CPB, the chest was closed in a standard manner with steel wires. In cases of an acute RHF, short-term right ventricular assist device (ST-RVAD) was implanted either through the right atrium and the pulmonary artery or via right jugular vein with a ProtekDuo^®^ cannula as previously described by Ruhparwar et al.^10^

### Anticoagulation protocol

After the ECLS implantation, the target activated clotting time (ACT) was maintained over 150 s or partial thromboplastin time (PTT) between 60 and 80 s via intravenous heparin administration.

After 12 h post-LVAD implantation, when the chest tube drainage decreased to ⩽50 ml/h and the coagulation profile returned to normal (or near-normal) levels, intravenous heparin infusion was commenced to maintain an activated partial thromboplastin time between 60 and 80 s. Aspirin 100 mg once daily was commenced after extubation. After removal of the chest drains and upon starting oral medication, phenprocoumon was administered to maintain an INR between 2.0 and 2.5. Intravenous heparin administration was continued until the international normalized ratio (INR) target range was achieved.

### Statistical analysis

Statistical analysis, including regression analysis, was performed using IBM SPSS version 27 (IBM Corp., Chicago, IL, USA) and R software v.3.4.3 (R Foundation for Statistical Computing, Vienna, Austria). Data were tested for normality using the Shapiro-Wilk test. Continuous variables were expressed as medians (interquartile range, IQR) or as mean ± standard deviation. Categorical variables were expressed as frequencies and percentages. The distributions of the continuous variables were compared between the groups with the t-test in cases of normal distributions and with the Mann-Whitney *U* test if the distributions were not normal. A *p*-value of less than 0.05 was considered to indicate statistical significance. For plotting the survival curves and for computing the mid-term mortality we used the Kaplan-Meier method.

## Results

### Baseline characteristics

The mean age at surgery was 52.3 ± 12.0 years and 14.3% of the cohort was female. All patients presented with multiple comorbidities, as shown in Table 1. All patients were admitted in acute cardiogenic shock with INTERMACS profile I and underwent an emergency rescue ECLS implantation as a bridge to durable LVAD at our institution. All patients suffered from an end-stage heart failure because of either dilatative cardiomyopathy (51.4%), ischemic cardiomyopathy (45.7%), or toxic induced cardiomyopathy (2.9%), presenting with a mean ejection fraction of 18.2 ± 8.6%.

**Table 1.** Baseline characteristics.

Characteristics	*n* (%)
Major bleeding	
Need for re-thoracotomy	15 (42.9)
Major infection	15 (42.9)
Localized non-device infection	4 (11.4)
Driveline infection	1 (2.9)
Pneumonia	5 (14.3)
Sepsis	11 (31.4)
Respiratory failure, *n* (%)	27 (77.1)
Ventilation over 6 days post implant	27 (77.1)
Reintubation	6 (17.1)
Tracheostomy	18 (51.4)
VV-ECLS	1 (2.9)
Right heart failure, *n* (%)	22 (62.9)
Mild right heart failure	4 (11.4)
Moderate right heart failure	10 (28.6)
Severe right heart failure	8 (22.9)
ST-RVAD	5 (14.3)
Hepatic dysfunction, *n* (%)	5 (14.3)
Acute renal dysfunction, *n* (%)	
Dialysis <90 days	24 (68.6)
Neurological dysfunction, *n* (%)	
Ischemic stroke	2 (5.7)
Intracranial hemorrhage	3 (8.6)
Hemolysis	0
LVAD thrombosis	1 (2.9)
Thromboembolism	0
Inotropic support > 7 days	21 (60)
Inotropic support > 14 days	14 (40)

LVAD: left ventricular assist device; ST-RVAD: short-term right ventricular assist device; VV-ECLS: veno-venous extracorporeal life support.

### Intraoperative data

**Figure 1.**
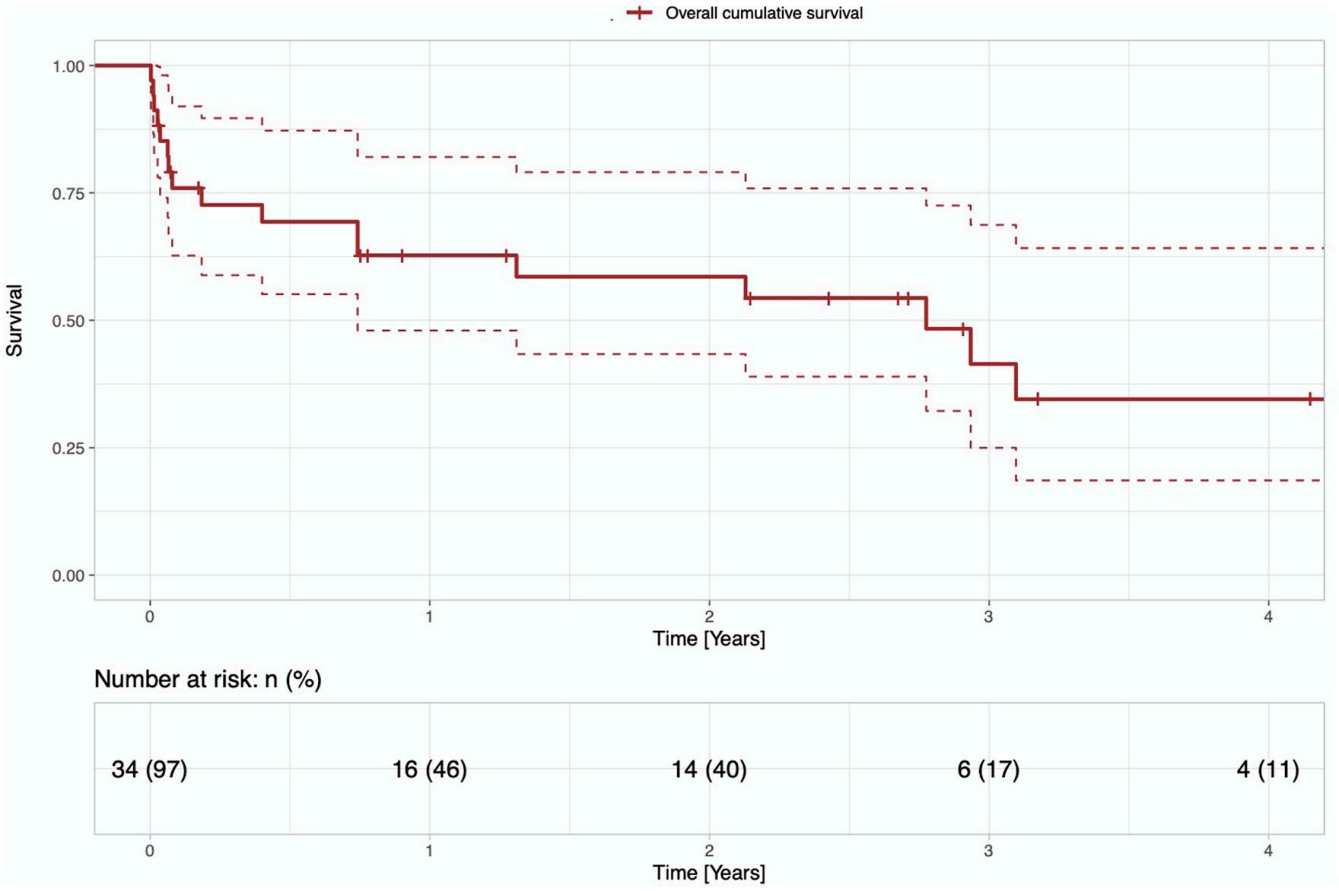
Overall survival.

The median length of ECLS bridge prior to LVAD implantation was 7 days (IQR 5.0–13). As shown in Table 2, patients underwent a rescue ECLS implantation for various indications. The intraoperative characteristics of LVAD implantation are portrayed in Table 3. Figure 1 shows the overall survival after the LVAD implantation. Weaning from ECLS or CPB after LVAD implantation was feasible in all patients.

**Table 2.** ECLS-related characteristics.

Characteristics	*n* (%)
Preoperative ECLS duration	7.0 (IQR 5.0–13.0)
ECLS concept	
Rescue ECLS	35 (100)
Peripheral ECLS	28 (80)
Central ECLS	7 (20)
Switch to central ECLS	6 (17.1)
Switch to Levitronix	7 (20)
Causes of cardiogenic shock	
Myocardial infarction	14 (40)
Cardial decompensation	17 (48.6)
Infection triggered	1 (2.9)
Other reason	3 (8.6)
Groin complications	8 (22.9)

ECLS: extracorporeal life support; IQR: interquartile range.

**Table 3.** Intraoperative characteristics.

Characteristics	*n* (%)
Duration, min	
Operation	237.1 ± 90.7
CPB	66.0 (IQR 0–127.0)
Plegia	3 (8.6)
LVAD model	
HeartMate III	6 (17.1)
HeartWare	29 (82.9)
Isolated procedure	27 (77.1)
Concomitant procedures	8 (22.9)
Tricuspid valve surgery	1 (2.9)
Aortic valve replacement	3 (8.6)
CABG	2 (5.7)
LAA closure	2 (5.7)

CABG: coronary arterial bypass grafting; LAA: left atrial appendage; LVAD: left ventricular assist device.

### Survival data and adverse events

The mean follow-up time was 0.8 years (IQR 0.1–2.8). In-hospital mortality was 34.3%, whilst 30-day mortality was 24.1%. After 6 months, 69.3% of patients survived, whilst after 1 year 62.7% (Table 4). During the hospital stay, 42.9% of patients underwent a re-sternotomy for bleeding, 42.9% developed a major infection, and 77.1% developed respiratory failure. RHF occurred in 62.9% of the patients and 14.3% of the patients needed a ST-RVAD implantation. About 14.3% of patients suffered a postoperative hepatic dysfunction and 68.6% of patients needed dialysis because of acute kidney failure. We also report an early LVAD thrombosis during the hospital stay in 2.9% of patients. The in-hospital adverse events are summarized in the Table 5. Of the cohort, 48.6% deceased during the follow-up period. One patient (2.9%) was able to be weaned from the LVAD support and three patients (8.6%) were successfully bridged to transplant. Driveline infection was in issue in 20% of the patients during follow-up, whilst another 20% of patients suffered LVAD thrombosis. The follow-up adverse events are summarized in Table 6. Table 7 shows the development of the laboratory findings prior to LVAD implantation and on the seventh postoperative day.

**Table 4.** Survival data.

Characteristics	*n* (%)
In-hospital mortality, %	12 (34.3)
Survival	
Follow-up time, years	0.8 (IQR 0.1–2.8)
30-day	75.9
6-months survival	69.3
1-year survival	62.7
Cause of death	
Cardiopulmonary failure	5 (14.3)
Infection	12 (34.3)
Cerebrovascular accident	4 (11.4)
Multiorgan failure	10 (28.6)
Bleeding	1 (2.9)
Unknown	1 (2.9)

**Table 5.** In-hospital major adverse events.

Characteristics	*n* (%)
Major bleeding	
Need for revision	15 (42.9)
Major infection	15 (42.9)
Localized non-device infection	4 (11.4)
Driveline infection	1 (2.9)
Pneumonia	5 (14.3)
Sepsis	11 (31.4)
Respiratory failure, *n* (%)	27 (77.1)
Ventilation over 6 days post implantation	27 (77.1)
Reintubation	6 (17.1)
Tracheostomy	18 (51.4)
VV-ECLS	1 (2.9)
Right heart failure, *n* (%)	22 (62.9)
Mild right heart failure	4 (11.4)
Moderate right heart failure	10 (28.6)
Severe right heart failure	8 (22.9)
ST-RVAD	5 (14.3)
Hepatic dysfunction, *n* (%)	5 (14.3)
Acute renal dysfunction, *n* (%)	
Dialysis <90 days	24 (68.6)
Neurological dysfunction, *n* (%)	
Ischemic stroke	2 (5.7)
Intracranial hemorrhage	3 (8.6)
Hemolysis	0
LVAD thrombosis	1 (2.9)
Thromboembolism	0
Inotropic support > 7 days	21 (60)
Inotropic support > 14 days	14 (40)

ST-RVAD: short-term right ventricular assist device; VV-ECLS: veno-venous extracorporeal life support.

**Table 6.** Follow-up outcomes and adverse events.

Characteristics	*n* (%)
Follow-up outcomes	
Death	17 (48.6)
Ongoing LVAD support	13 (37.1)
LVAD exchanged	2 (5.7)
Weaned from LVAD	1 (2.9)
Heart transplant	3 (8.6)
Follow-up adverse events	
Ischemic stroke	6 (17.1)
Intracranial hemorrhage	7 (20)
Thoracic bleeding	6 (17.1)
Gastro-intestinal bleeding	6 (17.1)
LVAD thrombosis	7 (20)
Driveline infection	7 (20)
Device malfunction	3 (8.6)
Right heart failure	6 (17.1)
Number of readmissions	0 (IQR 0–3.0)

**Table 7.** Laboratory parameters pre- and post-durable LVAD implantation.

Characteristics	Pre LVAD	7 days post LVAD	*p* Value
WBC	12.3 ± 5.7	14.2 ± 6.6	0.07
CRP (mg/l)	11.1 ± 6.2	12.2 ± 6.3	0.5
Creatinine (mg/dl)	1.4 ± 0.6	1.3 ± 0.7	0.94
Urea (mg/dl)	21.0 (IQR 1.1–42.0)	0.7 (IQR 0.5–2.03)	0.003
BUN (mg/dl)	4.2 (IQR 0.5–19.1)	0.3 (IQR 0.2–0.8)	0.004
Total bilirubin (mg/dl)	1.3 (IQR 0.7–4.9)	1.7 (IQR 0.75–3.2)	0.1
ALT (U/l)	43.0 (IQR 28.0–121.0)	43.0 (IQR 25.0–86.0)	0.24
LDH (U/l)	484.0 (IQR 400.0–610.0)	406.0 (IQR 348.0–550.0)	0.1
IL-6	38.5 (IQR 24.4–100.1)	55.75 (IQR 36.0–151.0)	0.33
PCT	1.0 (IQR 0.3–2.4)	0.8 (IQR 0.5–3.5)	0.68

ALT: alanine transmonase; BUN: blood urea nitrogen; CRP: C-reactive protein; IL: interleukin; LDH: lactate dehydrogenase; PCT: procalcitonin.

### Effects of ECLS bridge on the end-organ function

In Table 8, we demonstrate the laboratory findings that show a significant decrease in the kidney (creatinine and urea) and liver (total bilirubin, ASL, and ALT) parameters prior to LVAD implantation after the ECLS bridge. ECLS bridge allowed a significant improvement of the end-organ function.

**Table 8.** Laboratory parameters pre- and post-durable ECLS-bridge.

Characteristics	Pre ECLS	Pre LVAD	*p* Value
Creatinine, mg/dl	1.8 ± 0.6	1.35 ± 0.6	0.002
Urea, mg/dl	40.7 ± 18.7	21.0 (IQR 1.1–42.0)	<0.001
Total bilirubin, mmol/l	2.06 ± 1.8	1.3 (IQR 0.7–4.9)	0.014
ALT, U/l	182.0 (IQR 55.0–572.0)	43.0 (IQR 28.0–121.0)	0.056
AST, U/l	422.0 (IQR 62.0–1285.0)	61 (IQR 32.0–94–0)	0.003

ALT: alanine transaminase; AST: aspartate transaminase.

## Discussion

In the era of a significant organ shortage, liberal LVAD implantation expands the therapeutic options for patients presenting with an end-stage HF. Although there are no international guidelines for pre- and perioperative management of patients on long-term mechanical circulatory support (MCS), the number of implanted long- and -short-term MCS devices is growing rapidly.^11^ We conducted this study to enrich and expand upon the currently insufficient data on ECLS as a bridge to durable LVAD implantation in patients presenting with cardiogenic shock that is unrelated to postcardiotomy syndrome.

All the patients in our cohort were admitted with INTERMACS profile I, presenting as the most challenging group for surgical HF therapy with the highest mortality. Furthermore, without any alternative therapeutic option, it is likely that most of these patients would have died.^3^ Therefore, it is important to collect evidence in order to adequately develop surgical therapies for these patients, such that they are provided with an improved chance of survival.

In 80% of patients, ECLS was implanted via peripheral percutaneous groin cannulation. If a longer bridge duration was necessary for neurological assessment or because of the insufficient hemodynamic stabilization, the patients were switched to biventricular support with Levitronix (CentriMag, Thoratec) to provide a safer full hemodynamic support for a longer period of time. In fact, this concept was applied within 20% of the cohort. Zeriouh et al.^9^ described this method as feasible in patients with CS bridged to decision or to durable MCS. Unfortunately, for the implantation of biventricular Levitronix, a median sternotomy is necessary, leading to an increased risk of bleeding. On the other hand, Levitronix CentriMag was able to significantly reduce the RHF rates in the study of the Harefield group.

RHF is a significant complication in patients undergoing LVAD implantation, presenting in 20%–50% of all LVAD implantations.^12^ Zhigalov et al.^1^ described a 16.8% incidence of severe RHF after LVAD implantation in patients presenting with INTERMACS profile I and II. In our cohort, we report a relatively similar rate (22.9%) of severe RHF, albeit in an unhealthier group of patients presenting with CS and INTERMACS profile I, and further supported with ECLS prior to LVAD implantation. Therefore, similar to Schibilsky et al.,^7^ it is reasonable to postulate that ECLS therapy reduced the RHF rates in this significantly ailing cohort. Additionally, Schibilsky et al. described the importance of the ECLS weaning shortly before starting LVAD therapy to prevent the low-flow events. As a result, all our patients were weaned from ECLS or CPB prior to starting LVAD therapy. However, in 14.3% of patients a short-term RVAD (ST-RVAD) implantation was still necessary.

In 22.9% of patients we observed vascular groin complications after percutaneous cannulation, which is consistent with current literature.^13^ Therefore, we have since switched to ECPELLA 2.0 concept, allowing for full biventricular support without endangering the groin vessels,^10^ which, unfortunately, was not available at our institution at the time period that is assessed in this study. It should be noted that both Levitronix and ECPELLA 2.0 concepts are unsuitable for patients who are not hemodynamically stable. In such cases, a peripheral percutaneous bedside approach is most feasible.^14^

Three patients (8.6%) in our cohort underwent LVAD implantation whilst in cardioplegic arrest (as a result of a concomitant aortic valve procedure) and the remainder on the beating heart being either on CPB or with ECLS support. In fact, there exists an ongoing debate about the LVAD implantation on CPB or on veno-arterial ECLS (VA-ECLS) in patients bridged to durable LVAD. Currently, there is an absence of evidence suggesting an inferiority of the implantation on CPB. Moreover, it should be noted that Abdeena et al.^15^ in their comparative study, report of a non-inferiority of LVAD implantation on VA-ECLS in patients bridged to LVAD with ECLS.

Left-ventricular (LV) unloading is crucial for the outcomes in patients with ECLS as it prevents pulmonary edema and RHF. Schmack et al.^16^ in their single center trial, demonstrated and emphasized the importance of the LV decompressing and showed the near-significant superiority in the survival of patients on ECLS with LV-vent. Weymann et al.^17^ reported on the safe use of VA-ECLS with LV-unloading in patients with CS as a bridge to implantation of a biventricular assist device. Precise positioning of the venous ECLS cannula on the peripheral ECLS support is, therefore, of great importance. In the assessed period of time at our institution, in the cases where LV-decompression was required, a switch to biventricular Levitronix was performed. Currently, we tend to use the ECMELLA or ECPELLA 2.0 concept to ensure the LV-unloading of patients on ECLS, which has proven to be less invasive and very adequate, but often impossible in the setting of greater hemodynamic instability in acute CS.^10,18^

Within the period of 7 years that this study is covering, a significant shift from HVAD to HM III dominance can be observed over the years. Overall, 82.9% of our cohort underwent HVAD implantation, whereas after 2019, all patients (17.1%) were bridged to HM III device. Nevertheless, controversy surrounds this area as there is no clear evidence of superiority of any LVAD device.^19–21^ Theoretically, the prevalence of an older HVAD device in our study might be considered a study limitation.

Poor outcomes in patients bridged with MCS to durable LVAD have been reported by Boyle et al.,^3^ in line with the similar findings of Zhigalov et al.^6^ in their study of 20 patients in CS who, after MCS bridge, underwent a durable LVAD implantation. In the current cohort, we report a 30-day mortality of 24.1%, which is relatively acceptable and in line with literature, especially as patients with end-stage HF presenting in CS carry an extremely high mortality risk which, without mechanical support, have a nil to minimal chance of survival.^22^

Furthermore, whilst the overall mid-term mortality in our cohort was higher than in the INTERMACS registry, it can be explained by the few proportion of critically ill patients within the registry; 15.2% were admitted with INTERMACS profile I and of those, 20% needed ECLS bridge therapy.^23^ These critically ill patients were deliberately the only focus of our study in order to better evaluate the outcomes in this high-risk cohort. In our cohort 62.7% of patients were still alive after 1 year, demonstrating a real feasibility of the described surgical concept for this group of patients.

Consistent with Schibilsky et al.^7^ our findings presented a significant decrease in liver and kidney parameters on ECLS prior to LVAD implantation, portraying the recovery of the end-organ function prior to durable assist device implantation. In their study, Tsyganenko et al.^24^ describe liver and kidney failure to be independent mortality predictors in patients undergoing LVAD implantation with a prior bridge with ECLS. Therefore, it is crucial to stabilize the end-organ function prior to durable LVAD implantation and in this study, we demonstrate that rescue ECLS is able to provide the following.

### Study limitations

The retrospective nonrandomized nature of the study coming from a single center with a limited number of patients may have an impact on the outcomes and the study power, and can leave room for bias.

## Conclusions

The concept of bridging the patients presenting in CS with ECLS prior to durable LVAD implantation is a feasible approach to ensure acceptable survival rates and significant recovery of the end-organ function. Due to the rapid development of MCS technology, further studies with more modern and minimally invasive concepts of mechanical support as a bridge to durable LVAD implantation are required to support the current state of the literature.
